# Effect of neoadjuvant chemoradiation and postoperative radiotherapy on expression of heat shock protein 70 (HSP70) in head and neck vessels

**DOI:** 10.1186/1748-717X-6-81

**Published:** 2011-07-11

**Authors:** Frank Tavassol, Horst Kokemüller, Rüdiger Zimmerer, Nils-Claudius Gellrich, André Eckardt

**Affiliations:** 1 Department of Oral and Maxillofacial Surgery, Hannover Medical School, Hanover, Germany

## Abstract

**Background:**

Preoperative radiotherapy and chemotherapy in patients with head and neck cancer result in changes to the vessels that are used to construct microsurgical anastomoses. The aim of the study was to investigate quantitative changes and HSP70 expression of irradiated neck recipient vessels and transplant vessels used for microsurgical anastomoses.

**Methods:**

Of 20 patients included in this study five patients received neoadjuvant chemoradiation, another five received conventional radiotherapy and 10 patients where treated without previous radiotherapy. During surgical procedure, vessel specimens where obtained by the surgeon. Immunhistochemical staining of HSP70 was performed and quantitative measurement and evaluation of HSP70 was carried out.

**Results:**

Conventional radiation and neoadjuvant chemoradiation revealed in a thickening of the intima layer of recipient vessels. A increased expression of HSP70 could be detected in the media layer of the recipient veins as well as in the transplant veins of patients treated with neoadjuvant chemoradiation. Radiation and chemoradiation decreased the HSP70 expression of the intima layer in recipient arteries. Conventional radiation led to a decrease of HSP70 expression in the media layer of recipient arteries.

**Conclusion:**

Our results showed that anticancer drugs can lead to a thickening of the intima layer of transplant and recipient veins and also increase the HSP70 expression in the media layer of the recipient vessels. In contrast, conventional radiation decreased the HSP70 expression in the intima layer of arteries and the media layer of recipient arteries and veins. Comparing these results with wall thickness, it was concluded, that high levels of HSP70 may prevent the intima layer of arteries and the media layer of vein from thickening.

## Background

### Irradiation and vessels

The therapy of patients suffering from oral cancer could be recently improved by utilization of multimodal interdisciplinary regimes using a combination of surgery, chemo- and radiotherapy [1,2]. Extensive tissue defects following ablative tumor therapy do require adequate and functional reconstruction regardless of whether the patient received preoperative irradiation or not. During the last 20 years, the free vascularised tissue transfer became to be the "criterion standard" for reconstruction in head and neck cancers [3-6]. Large patient series with successful free flap transfer for head and neck reconstruction have been reported by many authors and demonstrated today's role as principal reconstructive procedure [5-10]. Since Guelinckx (1984) we know that irradiation of the recipient vessels in head and neck free flaps is leading to morphological changes [11]. Following studies confirm these results [12-14]. Different authors conclude, thus, although success is certainly possible when irradiated vessels are used for flap revascularization, there may be an increased risk of thrombosis, particularly in the head and neck [15,16]. Reviewing the current literature is leading to a different success rate of free flaps in irradiated patients ranging from 88% to nearly 100% [17-22]. Regarding the histological findings after irradiation, qualitative changes of the vessels such as hyalinosis of the intima and the media are described in literature [11,23]. Schultze-Mosgau et al. (2002) could show qualitative and quantitative histological changes to the recipient arteries, but not to the recipient veins following irradiation with 60-70 Gy. In contrast, neoadjuvant chemoradiation did not show changes to the recipient vessels [14].

### Heat shock proteins (HSP)

HSP are found in all organisms and all cell types. They are the most phylogenetically conserved proteins known with respect to both structure and function [24]. Usually, HSP are expressed at low levels, and under normal physiological conditions, many members of the HSP family are involved in protein synthesis. When a cell is stressed, oligomeric complexes disassemble and polypeptides unfold. Under these conditions, the role of HSP is to reverse such changes and, if refolding becomes impossible, to potentially speed up the removal of such denatured proteins. Expression of HSP is induced even under nonstress conditions, including those of the cell cycle, development, and differentiation [24-26]. Regarding the literature, even radiation could induce stress proteins in vitro [27]. Hurwitz et al. (2010) could show that radiation therapy induces expression of HSP70 in patients with prostate cancer [28]. Furthermore, the expression of heat shock proteins is induced by anticancer drugs such cisplatin [29,30].

### Aim of the study

The aim of the study was to investigate quantitative changes and HSP70 expression of irradiated neck recipient vessels and transplant vessels used for microsurgical anastomoses in free flaps in patients undergoing preoperative radiotherapy or neoadjuvant chemoradiation. The second aspect was to find out if HSP 70 might protect the transplant and recipient vessels.

## Methods

### Patients

The ethical approval was given by the local ethical committee. Of 20 patients included in this study (March 2004 - October 2006), 10 patients where treated without previous radiotherapy (group 1), five patients received conventional radiotherapy (59.4 - 72 Gy) (group 2) at least 22 months before surgery and another five patients received neoadjuvant chemoradiation 6 weeks before surgery (group 3, Table 1). The neoadjuvant chemoradiation protocol included cisplatin 12.5 mg/m^2 ^plus 40 Gy radiation or paclitaxel 40 mg/m^2^/carboplatin AUC 1.5 plus 40 Gy radiation [1,2]. During surgical procedure, 5-10 mm long vessel specimens where obtained by the surgeon. In each case a transplant artery and transplant vein from the raised flap and a recipient artery (superior thyroid or facial artery) and a recipient vein (facial vein) from the neck were achieved.

**Table 1:** Clinical data from patients included in the study

case No.	age	gender	pTNM	radiation dose (Gy)	time between radiation and surgery (months)	donor site	chemotherapy	neo adjuvant therapy	smoker
1	49	m	pT2 pN1	no radiation	------	radial forearm flap	-----		yes
2	74	m	pT4a pN2b	no radiation	------	radial forearm flap	-----		no
3	51	m	pT4a pN0	no radiation	------	fibula flap	-----		yes
4	84	f	pT2 pN0	no radiation	------	radial forearm flap	-----		no
5	69	f	pT4 pN2	no radiation	-----	radial forearm flap	-----		no
6	46	m	pT2 pN0	no radiation	------	radial forearm flap	-----		yes
7	65	m	pT4 pN0	no radiation	-----	latissimus dorsi flap	-----		yes
8	44	m	pT1 pN0	no radiation	-----	radial forearm flap	-----		yes
9	64	m	pT1 pN0	no radiation	-----	radial forearm flap	-----		yes
10	46	m	pT2 pN1	no radiation	-----	lateral arm flap	-----		no
11	55	f	ypT1 pN0	40	1,5	radial forearm flap	carboplatin + taxol	yes	yes
12	48	m	ypT1 pN0	40	1,5	latissimus dorsi flap	carboplatin + taxol	yes	yes
13	43	m	ypT1 ypN1	40	1,5	radial forearm flap	cisplatin/5-FU	yes	yes
14	55	m	ypT1 ypN0	40	1,5	radial forearm flap	carboplatin + taxol	yes	yes
15	57	m	ypT1 ypN1	40	1,5	latissimus dorsi flap	cisplatin/5-FU	yes	yes
16	57	m	pT4 pN3	60	108	radial forearm flap	-		yes
17	64	m	pT4 pN1	60	120	latissimus dorsi flap	-		no
18	30	m	pT2 pN2b	59.4	22	latissimus dorsi flap	-		no
19	51	m	pT4 N2b	61.2	20	latissimus dorsi flap	-		yes
20	58	m	pT4 N2a	72	81	radial forearm flap	-		no

### Immunhistochemistry

Serial 3-mm sections were deparaffinized, rehydrated, washed and, treated with a solution of 2% horse serum, 0.1% bovine serum albumin (Sigma Corporation, Steinheim, Germany), and 0.1% sodium acid in 150 mmol/l phosphate-buffered saline (PBS; pH 7.2) for 15 min to block nonspecific antibody-binding. A polyclonal rabbit anti-HSP70 antibody (Dako, Carpinteria, CA, USA), specific to HSP from Escherichia coli, which shares more than 48% sequence homology with mammalian HSP70 was the first layer. The optimal dilution of anti-HSP antibody (1:250) was determined by titration. The selected sections were incubated with this antibody for 120 min at room temperature (RT). The second layer, a biotin-conjugated goat antirabbit immunoglobulin (Oncogene, San Diego, CA USA) diluted 1:200 in PBS was incubated for 30 min at RT. The third layer was an avidin-biotin-horseradish peroxidase complex (Dako) diluted 1:50 in PBS. Incubation was, as before, 30 min at RT. Sections were washed for 10 min in 2 changes of PBS between each layer. The color reaction was developed with a solution consisting of 0.05% 3,30- diaminobenzidine tetrahydrochloride (Sigma, St Louis, MO, USA), 0.03% nickel chloride (Sigma), and 0.01% hydrogen peroxide in 48 mmol/l Tris-HCL, pH 7.6 (Sigma). Counterstaining was carried out with Mayer's hematoxylin [24,26].

### Quantitative evaluation

For quantitative histomorphometric analysis, cross-sections were obtained from the middle third of the vessels and analyzed with the image processing and analysis program analysis 3.1^® ^(Soft Imaging System, Münster, Germany). The measurement included the vessel wall thickness differed by the intima and the media and was carried out three times by two examiners. The mean values thus obtained were used for the following analysis.

### Evaluation of HSP 70 expression

Light microscopy and analysis 3.1^®^, an image processing and analysis program, were used for evaluating HSP70 expression. Respectively the intima, media or adventitia region was defined as the region of interest (ROI) and the percentage of HSP70-positive staining was analyzed [26].

### Statistical analysis

Statistical analysis was performed on a SPSS 18 statistical package. The specimens were compared for differences in percentage of HSP 70 staining and thickness of the intima and media part of the vessels respectively. One way repeated measures Analysis of Variance was used to detect differences and correlations at p values less than 0.05.

## Results

### Clinical data

There where three women (15%) and 17 men included in this study. The mean age of all patients was 55.5 years (range 30 to 84, median 55, SEM 3.69). The mean age of group 1 (no irradiation) was 59.2 years (median 57.5), the mean of group 2 (conventional irradiation, 59.4-72 Gy) was 52 years (median 57) and of group 3 (neoadjuvant chemoradiation, 40 Gy) 51.6 years (median 55). The mean duration between conventional radiation (group2) and surgical treatment was 70.2 months (median 80). Thirteen patients stated to be smokers (65%). Reconstruction was performed by using the radial forearm flap in 12 cases (60%), latissimus dorsi flap in six cases and each one by fibula flap and lateral arm flap (5% each, table 1). All flaps were successful.

### Vessel wall thickness

**Figure 1:**
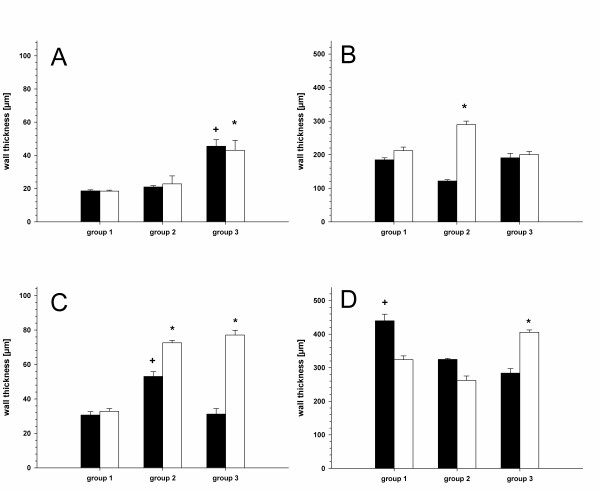
**Vessel wall thickness: patients without irradiation (group 1), patients with conventional irradiation (59.4-72 Gy, group 2) and patients treated with neoadjuvant chemoradiation (40 Gy and cisplatin or carboplatin and paclitaxel, group3)**. Intima of the veins (**A**, *; + p < 0.05 vs. group1 and group 2), media of the veins (**B**, * p < 0.05 vs. group 3), intima of the arteries (**C**, * p < 0.05 vs. group1, + p < 0.05 vs. group1 and group3) and media of the arteries (**D**, + p < 0.05 vs. group3, * p < 0.05 vs. group2). (black bars = transplant vessels; white bars = recipient vessels. Means and ± SEM).

The results of the vessel wall thickness are summarized in Figure 1A-D and table 2. The wall thickness of the vessels showed significant thickened intima layer of transplant and recipient veins in group 3 (neoadjuvant chemoradiation, Figure 1A). Conventional radiation (group2) led to thickening of the media layer of the recipient veins (Figure 1B). Regarding the arteries, conventional radiation (group2) and neoadjuvant chemoradiation (group3) revealed in a thickening of the intima layer of recipient vessels (Figure 1C). The media layer of the arteries led to a thickening of the recipient vessel in group3 (neoadjuvant chemoradiation) with a contemporary thinning of the transplant vessels in group2 (conventional radiation) and group3 (neoadjuvant chemoradiation, Figure 1D).

**Table 2:** Original data (mean ± SEM) for vessel wall thickness and HSP 70 expression (p < 0.05): patients without irradiation (group 1), patients with conventional irradiation (59.4-72 Gy, group 2) and patients treated with neoadjuvant chemoradiation (40 Gy and cisplatin or carboplatin and paclitaxel, group3)

	group 1		group 2		group 3	
**wall thickness [μm]**	*transplant*	*recipient*	*transplant*	*recipient*	*transplant*	*recipient*

intima of veins	18,6 ± 0,7	18,5 ± 0,6	21,0 ± 0,8	22,8 ± 4,8	45,6 ±4,0	43,1 ±6,1
media of veins	184,7 ±6,3	212,1 ±10,6	121,8 ± 3,9	289,6 ± 10,5	190,9 ± 13,2	200,5 ±9,3
intima of arteries	30,6 ± 2,0	32,8 ± 2,8	53,1 ± 2,8	72,5 ± 1,4	31,2 ± 3,2	77,1 ± 2,8
media of arteries	439,8 ± 19,6	323,6 ± 11,6	423,9 ±3,0	262,0 ±13,2	283,8 ± 13,5	405,5 ± 7,14

						
**HSP70 expression [%]**						

intima of veins	2,04 ± 0,18	3,06 ± 0,14	4,22 ± 0,14	2,54 ± 0,44	2,27 ± 0,15	2,92 ± 0,54
media of veins	2,42 ± 0,22	4,52 ± 0,21	2,46 ± 0,18	1,00 ± 0,05	4,55 ± 0,55	8,38 ± 0,94
intima of arteries	0,19 ± 0,01	1,56 ± 0,24	0,75 ± 0,23	0,77 ± 0,09	0,60 ± 0,12	0,35 ± 0,23
media of arteries	0,51 ± 0,07	2,41 ± 0,15	0,14 ± 0,01	0,32 ± 0,02	0,81 ± 0,19	4,41 ± 0,41

### HSP70 expression

**Figure 2:**
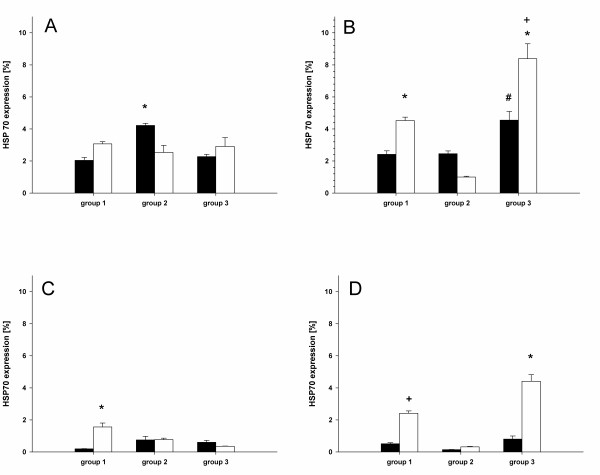
**Percentage of HSP70 expression: patients without irradiation (group 1), patients with conventional irradiation (59.4-72 Gy, group 2) and patients treated with neoadjuvant chemoradiation (40 Gy and cisplatin or carboplatin and paclitaxel, group3)**. Intima of the veins (**A**, * p < 0.05 vs. group1 and group 2), media of the veins (**B**, * p < 0.05 vs. group 2, + p < 0.05 vs. group 1 and **#**p < 0.05 vs. group1 and group2.), intima of the arteries (**C**, * p < 0.05 vs. group2 and group3) and media of the arteries (**D**, + p < 0.05 vs. group2, * p < 0.05 vs.group1 and group2). (black bars = transplant vessels; white bars = recipient vessels. Means and ± SEM).

**Figure 3:**
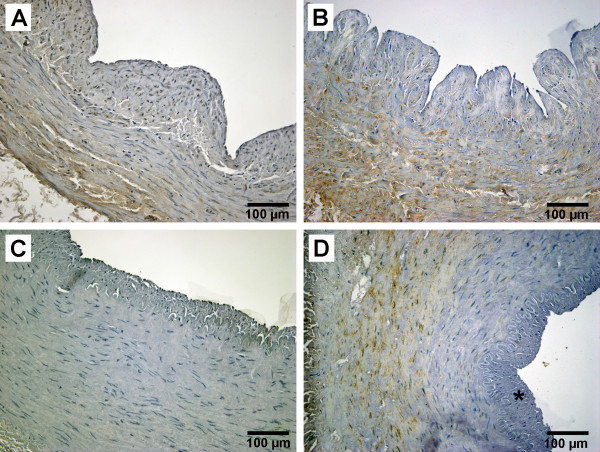
**Expression of heat shock protein (HSP) 70 in vessels of a patient treated with neoadjuvant chemoradiation: transplant vein (A), recipient vein (B), transplant artery (C) and recipient artery (D)**. (*) thickened intima;

The results of HSP70 expression are presented in Figure 2A-D and table 2. A increased expression of HSP70 could be detected in the media layer of the recipient veins as well as in the transplant veins of patients treated with neoadjuvant chemoradiation (group3) (Figure 2B, Figure 3A+B). Radiation (group2) and chemoradiation decreased the HSP70 expression of the intima layer in recipient arteries (Figure 2C). Regarding the arteries, an enhancement of HSP70 expression was limited to the media layer of the recipient vessels (group3, Figure 3D). Conventional radiation (group2) led to a decrease of HSP70 expression in the media layer of recipient arteries (Figure 2D, Figure 3C).

## Discussion

Today, free vascularised tissue transfer is the "criterion standard" for tissue reconstruction after ablative tumour therapy in head and neck oncology [3-6]. Many patients had been treated successfully in the last two decades [5-9]. For successful free tissue transfer, the quality of the transplant and the recipient vessels are desirable. Survival of free flaps is dependent on adequate blood supply. Pre-existing changes in transplant and recipient arteries may cause technical difficulties and must be regarded as additional factors contributing to graft failure [31,32]. Histopathologic damage of the recipient vessels in head and neck microsurgery can be caused by different reasons. Arteriosclerotic changes were often seen in patients suffering from head and neck cancer [31]. However, in many cases surgical treatment is not sufficient and adjuvant therapy might be necessary [1,2]. In these cases neoadjuvant chemoradiation therapy is used to increase local tumour control and to decrease the incidence of distant metastases. Nevertheless, a preoperative radiation is known to lead to histopathological changes in recipient vessels [12-14,14]. These morphological changes include hyalinosis of the intima and media layer and may increase the risk of thrombosis [15,16]. The current literature is describing different success rates of free flaps in irradiated patients ranging from 88% up to 100% [17-22]. A previous study from our department could demonstrate that neoadjuvant chemoradiation influenced the outcome of free vascularised tissue transfer while the circumstance if a patient is a smoker or not has no impact to success [6]. Therefore we did not discriminate between smokers and non smokers. To distinguish a possible effect of radiation from the influence of smoking in histomorphometry, we decided to harvest specimens from the transplant. The data of the present study showed different changes of the vessels influenced by preoperative radiation or chemoradiation therapy. A thickened intima layer of the recipient arteries in patients undergoing conventional radiation (group2, 59.4-72 Gy) or neoadjuvant chemoradiation (group3, 40 Gy) could be demonstrated. These findings are partially in contrast to the findings of Schultze-Mosgau et al. (2002), who described changes only after conventional radiation but not after chemoradiation [14]. The study of Schlutze-Mosgau et al. included a total of 93% smokers while our patient database contains only 60% smokers. Nevertheless, the chemoradation group (group 3) included 100% smokers is thus comparable. Another differing result is concerning the veins. We could demonstrate an enlargement of the intima layer of both, the transplant and the recipient vein. This could indicate that anticancer drugs may affect the veins especially considering that conventional radiation has no influence on the intima layer. Only the media layer is influenced by conventional radiation. We think this could be a long-time effect touching the recipient veins.

The second aspect of our study is concerning the HSP70 expression in vessels after radiation or chemoradiation. However, it is known, that radiation therapy or anticancer drugs can induce the expression of HSP70 [27-30,33,34]. Our findings can be concluded in three major results: 1. chemoradiation increases the HSP70 expression of the media layer in transplant and recipient veins while conventional radiation decreases the expression of HSP70 in the recipient vein. 2. conventional radiation and chemoradiation decreases HSP70 expression in the intima layer of recipient arteries, and 3. conventional radiation decreases HSP70 expression in the media layer of the recipient artery. Comparing the results of the intima layer of the arteries between HSP70 expression and wall thickness, it seems that low expression of HSP70 correlates with thickened intima layer. This applies to be the same in the media layer of the recipient veins. However, regarding these results it has to be considered that the sample power of this study is low and further study may be helpful to confirm these results.

## Conclusions

In the present study we could demonstrate, that radiation therapy is affecting the histomorphology of recipient veins of patients suffering from head and neck cancer. Although, there was no failure in our patients, the thickening of the intima layer in recipient arteries may influence success of free vascularised tissue transfer [17-22]. However, anticancer drugs can lead to thickening of the intima layer of transplant and recipient veins. HSP70 expression is decreased by conventional radiation in the intima layer of arteries and the media layer of arteries and veins. Anticancer drugs by contrast increase the HSP70 expression in the media layer of the recipient vessels. Comparing these results with wall thickness, it was concluded, that there might be some coherence between high levels of HSP70 expression and the prevention of thickening of the intima layer of arteries and the media layer of vein from.

## Competing interests

The authors declare that they have no competing interests.

## Authors' contributions

FT, HK, RZ, NCG and AE conceived of the study and participated in its design and coordination. FT drafted the manuscript, carried out the immunohistochemistry and performed the statistical analysis. All authors read and approved the final manuscript.

## Funding

The article processing charges are funded by the Deutsche Forschungsgemeinschaft (DFG), "Open Acess Publizieren".
